# Editorial: Plasticity of immune cells in tumor microenvironment

**DOI:** 10.3389/fonc.2023.1160961

**Published:** 2023-02-17

**Authors:** Valentyn Oksenych

**Affiliations:** Institute of Clinical Medicine (Klinmed), University of Oslo, Oslo, Norway

**Keywords:** tumor microenvironment, glioblastoma, glioma, USP22, gastric cancer

Immune cells, cancer-associated fibroblast, and tumor cells, together with the extracellular matrix and factors, form a tumor microenvironment (1). Tumor microenvironment modulates various cancer therapy options and can be used for a cancer treatment prognosis. While cancer-associated fibroblasts rather support tumor cells, presence of immune cells differs between individual tumors even of the same type. For example, tumors with a significant proportion of immune cells (“hot tumors”) are more likely to be cured than tumors with less immune cells but more cancer cells and cancer-associated fibroblasts (“cold tumors”) (2). Cancer-associated fibroblasts and tumor cells can produce factors inhibiting immune cells (T lymphocytes, natural killer cells, dendritic cells, macrophages) in the tumor microenvironment (1, 3). Cytotoxic T cells, natural killers, dendritic cells, and certain types of macrophages contribute to eliminating tumors. The current Research Topic’s purpose was to collect publications on the immune cells in the context of the tumor microenvironment.

In an original research article, Chen et al. focused on the role of macrophages in glioma cancer. Glioma is a cancer type that originates in spinal cord or brain from the glial cells that normally surround nerve cells and support their functions. Gliomas are a leading cause of cancer death around the world. Surgery, chemotherapy, and radiation therapy can help treat gliomas, but the overall survival for low-grade glioma is nine years, and for glioblastoma it is thirteen months (4). This bioinformatic-based study examined the prognostic and predictive value of antigen presentation machinery (APM) signature (5) in gliomas. This signature showed promising results in predicting survival and tumorigenic factors in glioma patients. The signature-based risk score was independently validated in three external cohorts and was able to predict immunotherapy response. Furthermore, the risk score-derived calreticulin (a product of *CALR* gene) (6) was found to mediate the invasion and polarization of macrophages. In conclusion, APM signature-based risk score can help improve the clinical management of gliomas.

In another original research article, Du et al. presented their finding on the metabolic signatures and antitumor immune response in the context of head and neck squamous cell carcinoma. Immune checkpoint inhibitor-based therapy, such as related to anti-programmed death 1 (PD1), anti-programmed death-ligand 1 (PD-L1), and anti-cytotoxic T lymphocyte-associated protein 4 (CTLA-4), has improved cancer survival rates (7, 8). In head and neck squamous cell carcinoma (HNSCC), recurrent or metastatic patients have also seen significant survival benefits from this therapy (9). Here, a metabolic-related gene prognostic index (MRGPI) was constructed to predict outcomes and immunotherapy response in head and neck squamous cell carcinoma. Seven genes were identified from the TCGA and GEO datasets (*ADA, ACADL, AGPAT4, AMY2B, CKM, HPRT1, and PLA2G2D*), and patients with a low MRGPI score had better overall survival than those with a high score. Low MRGPI scores were associated with lower metabolic activities, active antitumor immunity, and more benefit from immunotherapy. High MRGPI scores correlated with higher metabolic activities, higher *TP53* mutation rate, lower immune response, and less benefit from immunotherapy. In conclusion, MRGPI is a promising indicator for distinguishing the HNSCC prognosis, metabolic, molecular, and immune phenotype and predict immunotherapy benefits.

In the third original research article, Wang et al. focused on the tumor-secreted lactate and found that it modulates glioblastoma progression by modulating response to interferon-gamma by tumor, cytotoxic T cells, macrophages and microglia. Glioblastoma is one of the most aggressive types of cancer, and it originates in brain. This study examined the relationship between lactate and glioblastoma. Over 1400 glioblastoma samples were analyzed and the results were validated with another set of data. Lactate was found to predict glioblastoma progression, modulate the tumor immune landscape, and influence cytotoxic T cells and the tumor’s response to interferon-gamma. Machine learning algorithms and cell-cell interaction suggested that cytotoxic T cells and microglial cells may communicate with the tumor through lactate. Based on the cluster model, potential compounds which may offer new treatments were also predicted. Overall, lactate contributes to the immune suppressive microenvironment in the context of glioblastoma.

In the review article, Guo et al. focus on the immune evasion and drug resistance mediated by USP22 in cancer. Immune evasion is the way cancer cells escape and hide from the immune system, which otherwise recognizes and eliminates them. USP22 is a ubiquitin-specific peptidase 22 protein associated with various pathological conditions (10, 11). USP22 is a deubiquitinase involved in various cancer development-related cellular processes, such as cell cycle arrest, cell proliferation, apoptosis, invasion, metastasis, and immunity. It also affects proteome, may stabilize programmed death ligand 1 (PD-L1), and regulates T-cell infiltration into tumors. USP22 was found to positively regulate stable FOXP3 expression and reduce tumor volume when ablated in Tregs. This suggests that USP22 may regulate tumor resistance to immunotherapy. This article summarizes the biological functions of USP22 in multiple signal transduction pathways during tumorigenesis, immune evasion, and drug resistance, and proposes combining USP22 with chemotherapeutic, targeted, and immunosuppressive drugs in cancer treatment.

In the second review article, Zhao et al. discuss targeting immune cells for gastric cancer therapies. Gastric cancer (GC) is a malignancy with a high mortality rate. Immunotherapy has brought survival benefits to GC patients, offering durable responses and lower toxicity than traditional therapies. This review introduces the roles of each immune cell in the GC tumor microenvironment and summarizes the current immunotherapy strategies, such as immune checkpoint inhibitors, adoptive cell therapy (ACT), dendritic cell vaccines, reduction of M2 tumor-associated macrophages (TAMs) and N2 tumor-associated neutrophils (TANs), and reprogramming of TAMs and TANs. The most widely used therapies are PD-1/PD-L1 and CTLA-4 antibodies and CAR-T in ACT, and these strategies have shown anti-tumor efficacy in solid and hematological tumors. Targeting other immune cells provides a new direction for the immunotherapy of GC, and some of these strategies have entered clinical trials.

Overall, based on the articles on the Research Topic, there are two major directions of cancer therapy development, which will likely be relevant during the next decade. First is a wider usage of methods engaging the immune system to fight cancer *via* various immunotherapeutic protocols. Second is a broader usage of information technologies, including advanced methods of statistics, machine learning, and artificial intelligence approaches.

## Author contributions

The author confirms being the sole contributor of this work and has approved it for publication.
